# Factors Influencing Recovery From Pediatric Stroke Based on Discussions From a UK-Based Online Stroke Community: Qualitative Thematic Study

**DOI:** 10.2196/49409

**Published:** 2024-04-16

**Authors:** Charlotte Howdle, William James Alexander Wright, Jonathan Mant, Anna De Simoni

**Affiliations:** 1 School of Clinical Medicine University of Cambridge Cambridge United Kingdom; 2 Department of Public Health and Primary Care University of Cambridge Cambridge United Kingdom; 3 Wolfson Institute of Population Health Queen Mary University of London London United Kingdom

**Keywords:** child, stroke, rehabilitation, barriers, facilitators, internet-based intervention, self-help group, thematic analysis

## Abstract

**Background:**

The incidence of stroke in children is low, and pediatric stroke rehabilitation services are less developed than adult ones. Survivors of pediatric stroke have a long poststroke life expectancy and therefore have the potential to experience impairments from their stroke for many years. However, there are relatively few studies characterizing these impairments and what factors facilitate or counteract recovery.

**Objective:**

This study aims to characterize the main barriers to and facilitators of recovery from pediatric stroke. A secondary aim was to explore whether these factors last into adulthood, whether they change, or if new factors impacting recovery emerge in adulthood.

**Methods:**

We performed a qualitative thematic analysis based on posts from a population of participants from a UK-based online stroke community, active between 2004 and 2011. The analysis focused on users who talked about their experiences with pediatric stroke, as identified by a previous study. The posts were read by 3 authors, and factors influencing recovery from pediatric stroke were mapped into 4 areas: medical, physical, emotional, and social. Factors influencing recovery were divided into short-term and long-term factors.

**Results:**

There were 425 posts relating to 52 survivors of pediatric stroke. Some survivors of stroke posted for themselves, while others were talked about by a third party (mostly parents; 31/35, 89% mothers). In total, 79% (41/52) of survivors of stroke were aged ≤18 years and 21% (11/52) were aged >18 years at the time of posting. Medical factors included comorbidities as a barrier to recovery. Medical interventions, such as speech and language therapy and physiotherapy, were also deemed useful. Exercise, particularly swimming, was deemed a facilitator. Among physical factors, fatigue and chronic pain could persist decades after a stroke, with both reported as a barrier to feeling fully recovered. Tiredness could worsen existing stroke-related impairments. Other long-standing impairments were memory loss, confusion, and dizziness. Among emotional factors, fear and uncertainty were short-term barriers, while positivity was a major facilitator in both short- and long-term recovery. Anxiety, grief, and behavioral problems hindered recovery. The social barriers were loneliness, exclusion, and hidden disabilities not being acknowledged by third parties. A good support network and third-party support facilitated recovery. Educational services were important in reintegrating survivors into society. Participants reported that worrying about losing financial support, such as disability allowances, and difficulties in obtaining travel insurance and driving licenses impacted recovery.

**Conclusions:**

The lived experience of survivors of pediatric stroke includes long-term hidden disabilities and barriers to rehabilitation. These are present in different settings, such as health care, schools, workplaces, and driving centers. Greater awareness of these issues by relevant professional groups may help ameliorate them.

## Introduction

A pediatric stroke is classified as either perinatal (occurring when the child is 20 weeks in gestation to 28 days after birth) or childhood (from 29 days after birth to 18 years). There are >400 cases of pediatric stroke in the United Kingdom annually [1]. Pediatric stroke can be a debilitating disease, leaving survivors and their families coping with persisting issues during their recovery. These problems evolve and become more numerous as the survivor grows up, with as many as 75% of families of a child with stroke having at least 1 unmet need [2].

There is limited literature on factors that impact recovery. Survivors of pediatric stroke often develop comorbidities. Studies have found that survivors have an increased incidence of the following: epilepsy [3-5], attention-deficit/hyperactivity disorder [3,5], depression [5], anxiety [4,5], and autism [6,7], compared with their nonstroke counterparts. Fatigue [8] and behavioral problems [4,9] also hinder recovery. Mental health support [10] and follow-up neuropsychological assessments [10,11] positively influence outcomes. Qualitative studies with parents revealed that many disabilities experienced by stroke survivors are *hidden* [9], resulting in them not receiving the support they need. 

There are also emotional barriers that impact recovery. Pediatric stroke negatively affects the family of a survivor, with reports of poor parental mental health [4,11], guilt [4,12,13], and uncertainty about the future [12]. Having good parental welfare is important—parents are the primary caregivers following their child’s stroke, and their well-being impacts their child’s recovery. Good family functioning [4] also facilitates recovery from childhood stroke. Parents reported that treating the child as “normal” aided recovery [14]. A supportive social network, from close family and friends [14] to the wider society [13], is important for both parents and children to facilitate recovery.

Certain social factors are known to impact recovery. Qualitative reviews asking about patients’ experiences of health care showed that after discharge, parents felt abandoned by professionals [9,12]. Health care services may not be flexible, they may be difficult to access, and parents may not know where they can access therapy for their child [12]. When health care staff could be approached, parents may not have known what questions to ask [15]. Clear communication with parents by medical professionals about the causes of the stroke and events occurring around the stroke has been identified as an important issue [4]. Patients have appreciated positivity from clinicians [14], close and ongoing medical support [14,15], involvement with goal-setting approaches [16,17], and continuity of care. There is limited medical awareness or literature to support parents during their child’s recovery from stroke [2,9,12]. Support from charities and both in-person and online community groups partly addresses this issue [4,13,15]. Accessing disability aid also appears to facilitate recovery from childhood stroke [15].

A growing body of literature supports the potential of online health communities to provide opportunities for individuals to share their personal experiences and learn from others with similar conditions [18,19]. These communities also serve as a reliable and novel source of information about patients’ unmet needs [20,21], complementing more traditional research methods [22,23].

There are an increasing number of studies that explore the barriers to and facilitators of recovery from pediatric stroke, considering that these factors may be numerous and long lasting as children have a long poststroke life expectancy. However, there are relatively few studies that characterize the long-term impact of stroke on children; currently, the longest time frame studied is 5 years after stroke [24]. In May 2017, the Royal College of Paediatrics and Child Health in the United Kingdom published *The Stroke in Childhood Clinical Guidelines*, which had a set of “research recommendations” that included “reviewing the complications that children experience” following pediatric stroke to assess the **“**rehabilitation needs of pediatric stroke patients” and evaluate the “long-term outcomes” of survivors of pediatric stroke [25]. This study aims to address these recommendations by characterizing what survivors of pediatric stroke report as the main barriers and facilitators to their recovery in a UK-based online stroke community. As a secondary aim, the study also explores whether these factors last into adulthood or not, whether they change, or if new factors impacting recovery emerge in adulthood.

## Methods

### Study Design

We conducted a qualitative thematic analysis on posts from a pediatric stroke population within a UK-based online stroke community.

### Setting

The analysis used the archived TalkStroke online community, a UK-based, moderated online community hosted on the Stroke Association website from 2004 to 2011. In total, the TalkStroke archive contains 22,173 posts written by 2583 unique usernames. A previous study by De Simoni et al [18] identified 58 usernames that posted about experiences of pediatric stroke, contributing to a total of 469 posts. We excluded some users: 2 after further analysis revealed that their age at stroke was >18 years and a further 4 users because their age at the time of posting was unknown. A sample of 52 users remained. The characteristics of the survivors of pediatric stroke, including demographics, employment, education, stroke type, initial impairments as well as impairments at the time of posting, support needs, and independence, were retrieved from the data set of a previous study [18].

### Data Analysis

A deductive approach was used to develop predetermined themes before the start of the analysis. Factors (barriers or facilitators) were split into 4 themes based on recommendations by the Clinical Guidelines for Stroke in Childhood [25]: medical, physical, emotional, and social.

All relevant posts were collected in an Excel (Microsoft Inc) spreadsheet. WJAW and CH read all the posts to become familiar with the data. Themes were further split into subcategories using a data-driven approach by applying thematic analysis, as described by Braun and Clarke [26]. Coding was discussed until agreement was reached and a final coding framework was agreed upon. Each individual post was considered on its own, outside the context of the thread to which the posts belonged.

To consider the potentially different perspectives of participants over time, the posts were split into 2 categories: whether the survivor of pediatric stroke was aged ≤18 years or >18 years at the time of posting. This provided an assessment of whether the factors impacting recovery were short-term or long-term factors. We defined “long-term factors” as those affecting participants into adulthood and “short-term factors” as those impacting patients from the time immediately following a stroke until they reach 18 years of age. Sometimes, third parties (ie, parents or a member of the wider family) wrote on behalf of the survivor of pediatric stroke.

In this analysis, the term recovery describes the improvement of any aspect of stroke-related impairment. Rehabilitation is defined in concordance with the definition of the World Health Organization: “interventions designed to optimize functioning and reduce disability in individuals with health conditions in interaction with their environment” [27].

### Patient and Public Involvement

A 22-year-old survivor of pediatric stroke (when aged 0 years) was contacted after the analysis was completed. The patient and public involvement (PPI) participant was an acquaintance of a coauthor’s friend and was approached informally. We obtained written consent for her contribution to the work to be included in the paper.

The initial results were written and sent to the PPI participant, who read and provided insightful comments that highlighted important subcategories and informed our *Discussion* section.

### Ethical Considerations

The Stroke Association collected the data from the archived TalkStroke forum and provided the data set, together with their permission, for analysis for research purposes. The data are stored through the University of Cambridge Clinical School’s Secure Data Hosting Service, with reference S0126—Stroke Needs & Exp. This analysis was assessed by the University of Cambridge institutional review board and exempted from ethics approval, provided that permission to use the data was granted by the Stroke Association. At the time of registering with the community, users agreed that their data would be public. However, to protect the identity and intellectual property of participants, this analysis does not use direct quotes; instead, quotes are paraphrased. A more detailed account of the ethics involved with research on the TalkStroke archives is described in the study by De Simoni et al [18].

## Results

### Participants’ Characteristics

In total, 79% (41/52) of users were aged ≤18 years at the time of participation, contributing a total of 273 posts; 21% (11/52) of users were aged >18 years and contributed 152 posts. The majority of data from the ≤18-year-old group were collected through third-party users (35/41, 85%). Of these, most were written by the mother of the survivor (31/35, 89%). Data from the >18-year-old group were reported firsthand by adult survivors of pediatric stroke (Table 1).

Among the 11 participants who were aged >18 years at the time of posting, 2 (18%) held a driving license, 1 (9%) was considering applying for one, and 1 (9%) stated they do not drive. In total, 4 (36%) participants were university graduates or attending university, 1 (9%) was in part-time employment, and 1 (9%) was in full-time employment.

**Table 1:** Characteristics of the TalkStroke online community participants as identified in the posts.

Sample characteristics		All participants (n=52)	Participants aged ≤18 y (n=41)^a^	Participants aged >18 y (n=11)^a^
**Age (y) at stroke (if survivor of stroke)**				
	Mean	7.3	6	12
	Median (range)	6 (0-17)	4 (0-17)	13 (0-17)
Total number of posts, n		425	273	152
**Number of posts per participant**				
	Mean	8.2	6.7	13.8
	Median (range)	3 (1-56)	3 (1-56)	13 (1-33)
**Identity of the person posting, n (%)**				
	Survivor of stroke	17 (33)	6 (15)	11 (100)
	Mother	31 (60)	31 (76)	0 (0)
	Other (aunt, family friend, or cousin)	4 (8)	4 (10)	0 (0)
**Sex of the survivor of stroke, n (%)**				
	Male	24 (46)	19 (46)	5 (45)
	Female	26 (50)	20 (49)	6 (55)
	Not stated	2 (4)	2 (5)	0 (0)
**Time since stroke (y), n (%)**				
	0<x^b^<1	20 (38)	20 (49)	0 (0)
	1≤x<2	5 (0)	5 (12)	0 (0)
	>2	27 (52)	16 (39)	11 (100)
	Not stated	0 (0)	0 (0)	0 (0)
**Participants who stated they were, n (%)**				
	Holding a driving license	2 (18)	N/A^c^	2 (18)
	Not holding a driving license	2 (18)	N/A	2 (18)
	At university or some university	4 (36)	N/A	4 (36)
	In full-time employment	1 (9)	N/A	1 (9)
	In part-time employment	1 (9)	N/A	1 (9)

^a^Age at time of participation in the online stroke community.

^b^x: time or age between the 2 values.

^c^N/A: not applicable.

### Causes of Stroke and Resulting Impairments

Of the 52 survivors, 13 (25%) were survivors of right-sided strokes, 22 (42%) were survivors of left-sided strokes, 2 (4%) were survivors of stroke affecting both sides, and 15 (29%) were not reported. The causes of stroke were reported for a small number of participants: 4 (8%) were after surgery (3/4, 75% was due to cardiac operations and 1/4, 25% was unknown); 6 (11%) were after infection (1/6, 17% was meningitis; 3/6, 50% was chicken pox; 1/6, 17% was herpes encephalopathy; and 1/6, 17% was maternal shingles during gestation); 2 (4%) were ischemic; 3 (6%) were hemorrhagic; 1 (2%) was linked to acute lymphoblastic leukemia treatment; 2 (4%) were dissections; 1 (2%) was linked to a brain tumor; 1 (2%) was linked to arterial stenosis; 1 (2%) was linked to a septal defect; 1 (2%) was linked to an oral contraceptive pill; and 30 (58%) were not reported.

The initial impairments after stroke included the following: hemiplegia, hemiparesis, poststroke epilepsy, visual disturbances, tiredness, increased emotions, dystonia, dysarthria, facial droop, memory impairments, headaches, and no impairments. The difference between initial impairments and residual impairments at the time of posting varied greatly for users. The time between stroke and participation in the community ranged from 2 weeks to 46 years.

### Themes

The subcategories of the 4 themes are presented in Table 2, which shows whether the factors are short term, mostly reported by parents discussing their children with stroke, or long term, as reported by adult survivors of pediatric strokes.

**Table 2:** Themes and subcategories of factors impacting pediatric stroke.

Theme		Barrier	Facilitator	Short term	Long term
**Medical factors**					
	Comorbidities	✓		✓	✓
	Medical interventions		✓	✓	
**Physical factors**					
	Fatigue	✓		✓	✓
	Pain	✓		✓	✓
	Neurological sequelae	✓		✓	✓
**Emotional factors**					
	Positivity		✓	✓	✓
	Grief and bereavement	✓		✓	✓
	Fear	✓		✓	
	Anxiety	✓		✓	✓
	Behavioral problems	✓		✓	✓
**Social factors**					
	Support network		✓	✓	✓
	Loneliness	✓		✓	✓
	Exclusion or bullying	✓		✓	
	Hidden disabilities	✓			✓
	Education	✓	✓	✓	
	Driving	✓		✓	
	Travel	✓		✓	
	Third-party support	✓		✓	✓
	Financial support	✓	✓	✓	✓

### Medical Factors

#### Comorbidities

Epilepsy and depression were most commonly mentioned. Parents found it tough to cope with the additional diagnosis of epilepsy alongside pediatric stroke, with some reporting seeing their children getting more ill rather than better:

A parent writes they were completely shattered as their child had already suffered so much.

Mother; participant 30; ≤18-year-old group; stroke at the age of 11 years; 2 years since the stroke

One survivor wrote their depression sets them in a really low state of mind, where they cannot control their emotion.

Survivor; participant 47; ≤18-year-old group; stroke at the age of 15 years; 1 year since the stroke

Another, 35 years after their stroke, queried if it was possible to still feel depressed due to the stroke.

Survivor; participant 36; >18-year-old group; stroke at the age of 0 years; 35 years after the stroke

#### Medical Interventions

The survivors credited physiotherapy and speech therapy with helping them regain functionality and speech, respectively. The only downside mentioned was long waiting times to access services:

One parent advised others to get on top of physio immediately, saying that she went privately as waiting lists were long.

Mother; participant 12; ≤18-year-old group; stroke at the age of 1 year; 11 years after the stroke

One adult survivor writes that having physiotherapy sessions helped her walk.

Survivor; participant 36; >18-year-old group; stroke at the age of 0 years; 35 years after the stroke

Exercise was an activity recommended by medical practitioners that aided recovery. Parents and survivors mentioned the importance of repeatedly doing both general exercise and muscle-specific exercises to prevent muscle wasting, build muscle strength, help induce sleep, and increase fitness. Comments from the >18-year-old group also supported the utility of exercise, stressing the importance of regular daily activity. Swimming was written about consistently; it was recommended by parents as a way to get their children’s limbs to move, and it was noted that gains in swimming ability became an indicator of recovery and a source of excitement for the survivors and their families:

One individual advised parents to make a child do their exercises every day.

Survivor; participant 52; >18-year-old group; stroke at the age of 17 years; 21 years after the stroke

One parent wrote that swimming is a great way of exercising and moving their child’s limbs without trauma.

Mother; participant 11; ≤18-year-old group; stroke at the age of 1 year; 1 year after the stroke

Another wrote their child had 1 to 1 swimming lessons and could nearly do breaststroke again.

Mother; participant 25; ≤18-year-old group; stroke at the age of 8 years; 0 years after the stroke

Other medical interventions that the participants mentioned were the use of Botox for tightness in muscles. Although it was often useful, it did not always help. In addition, the participants aged >18 years endorsed the use of SaeboFlex [28] for regaining functionality, tai chi for mental health, and quinine for muscle cramps and spasms.

### Physical Factors

#### Fatigue

Fatigue was the most commonly reported physical barrier to recovery, with some parents writing that it was their main concern. When sleep duration was reported, it was at least 12 hours each night. The users told each other that tiredness was often caused by the large effort that the survivors were putting into their recovery. Fatigue was also a long-term factor:

A parent advised another that tiredness as a result of stroke is normal.

Mother; participant 26; ≤18-year-old group; stroke at the age of 9 years; 0 years after the stroke

One user reported that there was underlying tiredness throughout middle age, when they were working full time. In another post, they wrote that having a stroke resulted in less stamina, tolerance and energy in the context of noisy, busy backgrounds.

Survivor; participant 45; >18-year-old group; stroke at the age of 15 years; 46 years after the stroke

When a survivor became tired, this caused a worsening in disability. This affected speech, movement, and coordination:

A parent reported that when their child became tired on holiday, they were sad to see that their leg and arms were dragging.

Mother; participant 12; ≤18-year-old group; stroke at the age of 1 year; 0 years after the stroke

#### Pain

Pain was another commonly cited barrier preventing people from feeling fully recovered. Sites of pain were varied, including back pain, headaches, and limb muscle cramps. Pain emerged as a long-term consequence of a pediatric stroke. When the users discussed their pain with medical professionals, they were told that it was a result of their stroke and that it would ease with time. Being cold exacerbated chronic pain both in the short and long term:

One user reported that their headaches were seriously affecting their everyday life.

Survivor; participant 47; ≤18-year-old group; stroke at the age of 15 years; 1 year after the stroke

A user still has pain in their joints 35 years poststroke.

Survivor; participant 36; >18-year-old group; stroke at the age of 0 years; 35 years after the stroke

A parent wrote that their child’s affected limbs were more painful in the cold weather.

Mother; participant 32; >18-year-old group; stroke at the age of 13 years; 1 year after the stroke

#### Neurological Sequelae

Poststroke memory loss, confusion, and dizziness were the barriers to recovery both in the short and long term:

A parent described her child’s memory loss as causing as much trouble as the arm or foot.

Mother; participant 32; ≤18-year-old group; stroke at the age of 13 years; 1 year after the stroke

An individual 3 years poststroke commented that his eyes are dizzy.

Survivor; participant 41; >18-year-old group; stroke at the age of 11 years; 30 years after the stroke

### Emotional Factors

#### Positivity

Adult survivors of pediatric stroke stressed that positivity was an important factor in ensuring a successful recovery. It was mentioned less often by parents and younger survivors of pediatric stroke. However, humor was brought up by both groups as an important facilitator in coping with the consequences of stroke. Many possible fun activities were exchanged on the site. There was a large proportion of music-related activities, for example, singing songs with hand puppets:

One survivor stresses staying positive is the key to a successful recovery.

Survivor; participant 49; >18-year-old group; stroke at the age of 17 years; 2 years after the stroke

One parent writes their family tries to laugh themselves through bad times, rather than cry again.

Mother; participant 32; ≤18-year-old group; stroke at the age of 13 years; 1 year after the stroke

One parent wrote that music is a great mental stimulator as well as being fun.

Mother; participant 12; ≤18-year-old group; stroke at the age of 1 year; 0 years after the stroke

#### Grief and Bereavement

Following a pediatric stroke, the survivors wrote that they viewed themselves as different people and grieved for the person they once were. The families of survivors also commented that they experienced similar emotions and that the stroke had a long-term effect on the whole family:

One parent commented they have a different child now.

Mother; participant 31; ≤18-year-old group; stroke at the age of 11 years; 5 years after the stroke

Another survivor queried whether anyone on the forum felt that stroke was like a loss in the family and caused a grieving process.

Survivor; participant 51; >18-year-old group; stroke at the age of 17 years; 4 years after the stroke

One parent commented that they don’t think any of their family will ever be the same as before the stroke.

Mother; participant 32; ≤18-year-old group; stroke at the age of 13 years; 1 year after the stroke

#### Fear

Fear of uncertainty and the unknown was commonly cited as a barrier to recovery. Parents wrote that the fear of another stroke event, how their child will grow up and fit into society, and having no explanation of the cause of the stroke were particularly difficult issues. In contrast, adult survivors encouraged users to accept the stroke and not let fear stand in the way of recovery:

A parent wrote that it was hard to comprehend the unknown future.

Mother; participant 11; ≤18-year-old group; stroke at the age of 1 year; 1 year after the stroke

One adult survivor wrote that acceptance of stroke is the first stage of the healing process and survivors must move on and get on with their lives.

Survivor; participant 46; >18-year-old group; stroke at the age of 15 years; 28 years after the stroke

#### Anxiety

Both parents and survivors described the scenarios that caused upset by reminding them of the trauma surrounding the stroke event:

One parent writes that her daughter was admitted to hospital and it brings back too many memories which makes their calm slip a little inside.

Mother, participant 12; ≤18-year-old group; stroke at the age of 1 year; 0 years after the stroke

An adult survivor training to be a health care professional writes that having a placement on a stroke unit is difficult to cope with.

Survivor; participant 51; >18-year-old group; stroke at the age of 17 years; 4 years after the stroke

#### Behavioral Problems

Behavioral problems were commonly mentioned. Parents reported mood swings with negative emotions, such as getting upset, anger, and frustration. There were multiple posts trying to rationalize why these changes occur, possible reasons being parental spoiling of the child either before or after the stroke and typical teenage mood swings. Adult survivors of pediatric stroke mentioned some lasting behavioral problems:

A parent wrote that their child gets upset with how she feels and that her brain seems to tell her things that she can’t cope with.

Mother; participant 30; ≤18-year-old group; stroke at the age of 11 years; 2 years after the stroke

One survivor wrote that he takes himself to bed when he gets really grumpy.

Survivor; participant 52; >18-year-old group; stroke at the age of 17 years; 21 years after the stroke

Another survivor queries whether it is possible to have mood swings and feel low 35 years after a stroke as she is struggling.

Survivor; participant 36; >18-year-old group; stroke at the age of 0 years; 35 years after the stroke

### Social Factors

#### Support Network

Parents stressed the importance of supporting their children throughout the stroke recovery process. Support was appreciated by the survivors of stroke, who reiterated the importance of love and support in motivating them during recovery:

One parent wrote *it is completely down to the family to get their child through recovery.* [Mother; participant 12; ≤18-year-old group; stroke at the age of 1 year; 0 years since the stroke]

An adult survivor of pediatric stroke wrote that they fought every inch of the way with the love and support of their parents.

Survivor; participant 39; >18-year-old group; stroke at the age of 8 years; 23 years since the stroke

A user mentioned their friend by name and thanked them for getting them over the mental side of things.

Survivor; participant 42; >18-year-old group; stroke at the age of 13 years; 7 years after the stroke

#### Loneliness

Loneliness emerged in many contexts as both a short-term and a long-term barrier to recovery from stroke. Loneliness made the survivors feel different from those around them, which negatively impacted their well-being. The survivors felt isolated from fellow survivors of stroke, friends, family, and peers:

A male survivor wrote he struggled to find any help and support with his rehabilitation.

Survivor; participant 43; >18-year-old group; stroke at the age of 13 years; 7 years after the stroke

A female survivor commented she never talks about her stroke to her friends because she doesn’t want to be judged.

Survivor; participant 36; >18-year-old group; stroke at the age of 0 years; 35 years after the stroke

The parent of a survivor writes that her child’s class look at him differently.

Mother; participant 20; ≤18-year-old group; stroke at the age of 3 years; 2 years after the stroke

Loneliness had long-lasting effects on survivors of stroke:

One member of the >18 group reported they felt alone most of their life. They then go on to tell a user they have done the best thing joining the site.

Survivor; participant 36; >18-year-old group; stroke at the age of 0 years; 35 years after the stroke

#### Exclusion or Bullying

There were reports of bullying and exclusion behaviors among children at school. Furthermore, parents reported that social restrictions, such as not allowing the survivors to ride a bicycle or go out with friends, were a barrier to recovery:

A survivor’s mother wrote that her daughter is taunted at school by her own friends for having a stroke.

Mother; participant 26; ≤18-year-old group; stroke at the age of 9 years; 0 years after the stroke

One parent wrote that the restrictions placed on her son prompted him to tell them that he may as well be dead.

Mother; participant 22; ≤18-year-old group; stroke at the age of 5 years; 4 years after the stroke

#### Hidden Disabilities

Being doubted about their degree of disability was reported by several adult survivors of pediatric stroke. They described the medical staff and the public as not understanding the hidden effects of stroke and assuming that if there are no visible impairments, they are recovered. This discredited the survivor’s struggle for recovery and made them feel upset:

A survivor wrote how she told a midwife about her stroke and the midwife’s response was that because they hadn’t noticed at first glance, it couldn’t have affected the survivor that badly. The survivor commented how angry that made her feel.

Survivor; participant 36; >18-year-old group; stroke at the age of 0 years; 35 years after the stroke

A survivor reported people discrediting their tiredness, accusing them of being selfish or lazy.

Survivor; participant 51; >18-year-old group; stroke at the age of 17 years; 4 years after the stroke

#### Education

There were mixed reviews about the education services accessed; however, interaction with the education sector was highlighted as important in getting the survivor of pediatric stroke back into school and therefore integrating back into society:

One parent writes that her child’s new school is not dealing with her needs very well, and they have had to go in on several occasions.

Mother; participant 32; >18-year-old group; stroke at the age of 13 years; 1 year after the stroke

#### Driving

There was concern from parents of survivors of stroke as to whether their children could drive. There was much reassurance and advice given by other parents and survivors in the >18-year-old group:

One parent writes that her child had his stroke at 11 and was then 16 and wondered if he would be able to drive.

Mother; participant 31; ≤18-year-old group; stroke at the age of 11 years; 5 years after the stroke

One survivor commented he overcame medical predictions and had been driving since the age of 21.

Survivor; participant 46; >18-year-old group; stroke at the age of 15 years; 28 years after the stroke

#### Travel

Parents reported that obtaining travel insurance for their child was difficult. Many companies did not provide insurance at all; they only insured per trip and not for longer periods as parents wanted, and the price of insurance was high, deterring the families from traveling:

One parent commented that they found it mind-blowing that they must pay so much and no one else will cover them.

Survivor; participant 52; >18-year-old group; stroke at the age of 17 years; 21 years after the stroke

#### Third-Party Support

The members often recommended external resources to each other. These were most often helplines or information on websites. Organizations that were mentioned were the Stroke Association [1], HemiHelp [29], Maypole project [30], Different Strokes [31], Mobilise [32], Disability Information and Advice Line [33], and Headway [34].

#### Financial Support

Financial support for disability was available for survivors of pediatric stroke and was identified as helpful. However, there was discussion among adult survivors of pediatric stroke who expressed fear of having financial support taken away from them:

One survivor wrote that they had read an article about how people with mental health issues are more likely to fail the assessment test, as they have multiple symptoms which vary and she was worried that this would disadvantage stroke survivors also.

Survivor; participant 45; >18-year-old group; stroke at the age of 15 years; 46 years after the stroke

### PPI Feedback

The analysis was read by a survivor of multiple childhood strokes while aged <1 year who is now studying at a university. She reported strongly agreeing with uncertainty about the future, the benefits of swimming, the hidden disabilities because of stroke, and the lack of public awareness of pediatric stroke. Some quotes are reported in the subsequent section to illustrate the PPI feedback:

- Regarding swimming:

I was also told it was important for me to swim to help prevent any damage as I developed so swam from 6 months on.

- Uncertainty about the future:

This strongly resonates with me and my family. For my parents it was a huge unknown as the doctors couldn’t tell them if I would be able to walk, speech or do well in school. They also couldn’t tell if I was going to have another one, but if I didn’t have another by 12 I should be clear from not having another.

- Hidden disabilities:

I would agree hidden effects of a stroke are not spoken about. In the main all I was warned of was physical disabilities and those which would be major hindrances in my life. By this I mean walking, talking, learning and doing physical exercise/sport well. Once it was clear at ~7 or 8 year old I was able to conduct those tasks no further check ups were taken and no-one checked for any other less major/obvious effects. As for the public, I would strongly agree many have no idea of the effects of strokes, even I didn’t realise tiredness was an effect until I read this paper! I have an absolutely terrible memory and I do think if this is very likely down to my stroke and this I would say is a hidden disability. I’m almost certain my stroke affected the memory side of my brain so it would make some sense. However, I assume few members of the public would understand this and no medical staff checked or mentioned smaller effects other than physical movement/speech/learning.

## Discussion

### Principal Findings

Medical, physical, emotional, and social themes were identified as impacting the recovery from pediatric stroke. Exercise, swimming, speech therapy, and physiotherapy were the medical factors that facilitated recovery, whereas having a comorbidity hindered recovery. A novel finding of this study is that “hidden” physical impairments, such as fatigue, pain, memory loss, confusion, and dizziness, affected survivors of pediatric stroke in the long term, with the lack of awareness of these impairments by the general public and by professionals in health care, school, and workplace settings also hindering recovery. Grief, fear of the future, restrictions on the child’s life, and behavioral problems were the emotional factors that slowed recovery, whereas positivity and a good support network aided recovery. Isolation, hidden disabilities, triggering events, finding travel insurance, and fear of not being able to drive were the social factors hindering recovery. Third-party support and financial aid were facilitators of recovery, albeit there was a fear of losing financial aid as survivors of pediatric stroke aged.

Identifying the factors that impact recovery from pediatric stroke is important, as there is the potential for survivors to live for many decades after the stroke, and therefore, they may live many years with disabling factors. Our study found that fatigue persists many decades after stroke, which expands on current literature that fatigue is an identified barrier up to 5 years after the stroke [8]. In addition, tiredness was reported to exacerbate stroke-related disabilities. This relationship has been reported in adult brain injury [35], but to the best of our knowledge, this is the first time it has been reported in pediatric stroke. In addition, this study is the first to report chronic pain as a barrier to recovery in pediatric stroke, despite chronic pain being well known because of adult stroke [36].

Rehabilitation therapies are recommended following pediatric stroke; however, there is little evidence that describes survivors’ experiences of these services. This study was the first to report that survivors of pediatric stroke found that both muscle-specific exercise and general exercise helped in recovery. The Royal College of Physicians national guidelines for adult stroke recommend muscle-specific exercises to improve functionality and found general exercise to help aerobic fitness, gait, and prevent regression of cognitive and functional gains after stroke as well as have positive psychological effects [37]. To our knowledge, no such research has established this for pediatric stroke. A new finding from this study was that swimming facilitated recovery. This has not been researched in relation to pediatric stroke, but a feasibility study on an introductory performance-focused swimming intervention for adult cerebral palsy found that swimming helped fatigue, physical function, and mental health [38]. Speech and language therapy is commonly used during rehabilitation, despite a lack of evidence [25]. This study provides qualitative evidence that participants found this intervention useful and recommended it to other users. Similarly, this is the first qualitative study to explore patients’ experiences of physiotherapy.

Our study found that stroke-related social restriction on a child’s life has a negative emotional effect. This is an important finding, as survivors of pediatric brain injuries, including stroke, commonly have lower levels of community activity and peer social play at school [39]. Another finding from this study was that isolation and exclusion were barriers to recovery. Denham et al [15] found that following a pediatric stroke, families feel abandoned by friends or their intimate support network. Our study further characterized this dimension as “isolation” from other survivors, friends, the wider family, and society. Bullying has also been found to be a barrier in previous studies [40,41]. Uncertainty about the future has also been reported [12]. This study found this is a short-term barrier, as participants in the >18-year-old group did not share this concern, instead putting the stroke behind them and being more positive about the future. Financial support is accessible after recovery from stroke. However, a new finding from this study was that there was a long-term fear of losing financial support as disability allowance was reassessed. Driving is an important indicator of independence and recovery after an adult stroke [42]. This study found that the parents and the survivors were concerned about whether they could drive after a pediatric stroke, and this concern was often expressed many years after the stroke. This was only a short-term concern, as the users were reassured by adult survivors of pediatric stroke who had driving licenses and could explain the process of learning to drive with a disability. Difficulty obtaining travel insurance has been reported so far only in the literature on adult stroke [43].

This study found that both parents and children experienced anxiety-triggering events that reminded them of the happenings of their original stroke. Although a diagnosis cannot be reached from these comments, Lehman et al [44] found increased symptoms of posttraumatic stress disorder in children and their parents, suggesting that the memory of stroke has long-lasting emotional effects on the family. Our findings support previous evidence that developing comorbidities [3-7], behavioral problems [4,9], and the bereavement process caused by the stroke [45] were factors hindering recovery. A good support network [13] and third-party support and information aided recovery [4,13,15], and the users often recommended useful resources to each other.

### Strengths and Limitations

The strength of this study lies in the source of data, which is a UK-wide online community with several survivors of pediatric stroke at varying stages in their lives. First, the community facilitated the discussion between participants who had a stroke recently and users who had a stroke many years ago. This allowed unprecedented insight into longer-term factors inhibiting recovery. Second, the discussions were initiated by the participants and continued in an asynchronous way, with no time, geographical, or behavioral constraints on communication. This dynamic cannot be replicated in traditional research approaches, for example, in interviews. Finally, the population that uses the forum might include people who do not partake in traditional research studies, thereby including perspectives from an underrepresented patient population [23]. The limitations of this study are that the users may not mention all the factors that affect their recovery; therefore, our analysis may not be comprehensive. First, this may be because the users do not raise everything pertinent to the research question, the users were only active over a limited period of their recovery, or the forum was moderated, so some posts may have been removed or affected by the moderation process. Second, the authenticity of posts could not be determined. Third, the data set is individuals aged >10 years, and factors affecting recovery may have changed in the time between the posts and this analysis. PPI feedback was limited to a single individual. Finally, there is a nonactive population that reads but does not compose messages. These users may be more numerous than the registered users themselves; one study [46] reported a 26:1 ratio of lurkers for every author of a message in an online forum. This population cannot be quantified or classified.

### Conclusions

This study identified novel findings regarding factors affecting recovery from pediatric stroke. Raising awareness about the lived experience of survivors of pediatric stroke and the type and impact of long-term impairments is needed in health care settings, schools, workplaces, driving centers, and travel agencies so that appropriate support and information can be provided. Recovery from a stroke is an evolving process that lasts decades. Although this study has highlighted some long-term factors impacting recovery, more research needs to be performed to further establish these as well as design interventions to alleviate these barriers. This will result in effective, long-term support for survivors. In particular, fatigue, pain, and loneliness were the physical problems that were present many decades after the stroke, and there are no effective interventions reported yet.

## Abbreviations

PPI: patient and public involvement
